# Establishment of Routine Clinical Indicators-Based Nomograms for Predicting the Mortality in Patients With COVID-19

**DOI:** 10.3389/fmed.2021.706380

**Published:** 2021-10-18

**Authors:** Jialin He, Caiping Song, En Liu, Xi Liu, Hao Wu, Hui Lin, Yuliang Liu, Qi Li, Zhi Xu, XiaoBao Ren, Cheng Zhang, Wenjing Zhang, Wei Duan, Yongfeng Tian, Ping Li, Mingdong Hu, Shiming Yang, Yu Xu

**Affiliations:** ^1^Huo-Shen-Shan Hospital, Wuhan, China; ^2^Jin Yin-tan Hospital, The Medical Team of the Army Medical University, Wuhan, China; ^3^Department of Gastroenterology, Xinqiao Hospital, The Army Medical University, Chongqing, China; ^4^Xinqiao Hospital, The Army Medical University, Chongqing, China; ^5^Taikang Tongji Hospital, Wuhan, China; ^6^Department of Respiratory and Critical Care Medicine, The First Affiliated Hospital of Chongqing Medical University, Chongqing, China; ^7^Department of Respiratory and Critical Care Medicine, Xinqiao Hospital, The Army Medical University, Chongqing, China; ^8^Department of Emergency, Xinan Hospital, The Army Medical University, Chongqing, China; ^9^Department of Hematology, Xinqiao Hospital, The Army Medical University, Chongqing, China; ^10^Department of Neurology, Xinqiao Hospital, The Army Medical University, Chongqing, China; ^11^Department of Endocrinology, Xinqiao Hospital, The Army Medical University, Chongqing, China; ^12^Department of Cardiology, Xinqiao Hospital, The Army Medical University, Chongqing, China

**Keywords:** COVID-19, mortality, prediction, nomogram, the MuLBSTA score

## Abstract

This study aimed to establish and validate the nomograms to predict the mortality risk of patients with coronavirus disease 2019 (COVID-19) using routine clinical indicators. This retrospective study included a development cohort enrolled 2,119 hospitalized patients with COVID-19 and a validation cohort included 1,504 patients with COVID-19. The demographics, clinical manifestations, vital signs, and laboratory tests of the patients at admission and outcome of in-hospital death were recorded. The independent factors associated with death were identified by a forward stepwise multivariate logistic regression analysis and used to construct the two prognostic nomograms. The nomogram 1 was a full model to include nine factors identified in the multivariate logistic regression and nomogram 2 was built by selecting four factors from nine to perform as a reduced model. The nomogram 1 and nomogram 2 showed better performance in discrimination and calibration than the Multilobular infiltration, hypo-Lymphocytosis, Bacterial coinfection, Smoking history, hyper-Tension and Age (MuLBSTA) score in training. In validation, nomogram 1 performed better than nomogram 2 for calibration. We recommend the application of nomogram 1 in general hospitals which provide robust prognostic performance though more cumbersome; nomogram 2 in the out-patient, emergency department, and mobile cabin hospitals, which depend on less laboratory examinations to make the assessment more convenient. Both the nomograms can help the clinicians to identify the patients at risk of death with routine clinical indicators at admission, which may reduce the overall mortality of COVID-19.

## Introduction

With the continuing pandemic of severe acute respiratory syndrome coronavirus 2 (SARS-CoV-2) infection, there have been more than 200 million patients with coronavirus disease 2019 (COVID-19) globally and more that 4 million deaths as of August 8, 2021. The clinical manifestations and outcomes of COVID-19 have been delineated in several studies, with 81% of patients presenting with subtle or minor symptoms, and 19% severe or critical cases (1). The mortality of COVID-19 was 11% as first reported (2), dropped to 2.3% (1,023 of 44,672 confirmed cases) on February 11, 2020, in China (3) and was reported to be 2.1% according to the data provided by WHO as of August 8, 2021. The previous studies have reported that the risk factors, such as age, pre-existing comorbidities, and neutrophil-to-lymphocyte ratio (NLR) were associated with higher mortality risk in COVID-19 (1, 4–7). During the initial outbreak of COVID-19 in Wuhan, the MuLBSTA score (8) was reported to be associated with the outcome of COVID-19 in a few observational studies (9). However, the MuLBSTA score was developed to assess the outcome of influenza A, rhinovirus, and other respiratory virus pneumonia and has not been rigorously tested in predicting the risk of death in patients with COVID-19. A nomogram, widely used output of the prognostic model, generate an individual probability of a clinical event with a visualized interface. There are few studies of nomograms in predicting the death risk of COVID-19 (10–13). The nomograms developed from those studies provided useful tools for the researchers and clinicians in stratifying patients with COVID-19. However, the size of participants enrolled was limited in all the above studies, and a lack of independent validation was noted in one study (10).

In this study, we aimed to (1) develop a full model (nomogram 1 designated as Nomo1) and a reduced model (nomogram 2 designated as Nomo2) with routine clinical indicators to predict the risk of death using 2,119 cases of confirmed COVID-19; (2) compare the predictive efficacy of Nomo1 and Nomo2 with the MuLBSTA score; (3) assess Nomo1 and Nomo2 in an external validation cohort comprising 1,507 cases.

## Materials and Methods

### Study Design and Participants

This retrospective study included a training cohort before being tested in a validation cohort. Within 10 days, from January 22 to February 2, 2020, an emergency hospital with 1,000 beds named Huo-Shen-Shan (HSS) hospital was built in Wuhan (Hubei Province, China) by the Chinese government to admit confirmed patients with COVID-19. In the training cohort, the patients admitted to HSS Hospital from February 4 to March 31, 2020, were retrospectively screened and were followed up to April 15, 2020, when the HSS Hospital closed. A validation cohort included the patients with COVID-19 admitted to Jin Yin-tan Hospital (Hubei Province, China) from January 26 to February 1, 2020 and the patients with COVID-19 admitted to Taikang Tongji Hospital (Hubei Province, China) from February 19 to April 2, 2020. In this way, we almost covered the whole spectrum of time from the COVID-19 outbreak to remission in Wuhan in the validation cohort to ensure the data are representative. The study followed the Transparent Reporting of a multivariable prediction model for Individual Prognosis Or Diagnosis (TRIPOD) checklist, and was approved by the ethics committee of Xinqiao Hospital (2020-yd073-01), Chongqing, China with written informed consent waived due to the retrospective nature of the study.

### Inclusion and Exclusion Criteria

#### Inclusion Criteria

The patients diagnosed according to the WHO interim guidance for COVID-19 were included in the study (14).

#### Exclusion Criteria

(1) Receiving cardiopulmonary resuscitation on admission and fail to survive; (2) the cases with missing data of some important demographics and clinical indicators; (3) duplications due to readmission or in-hospital transfer between wards.

### Procedure and Data Collection

**Figure 1:**
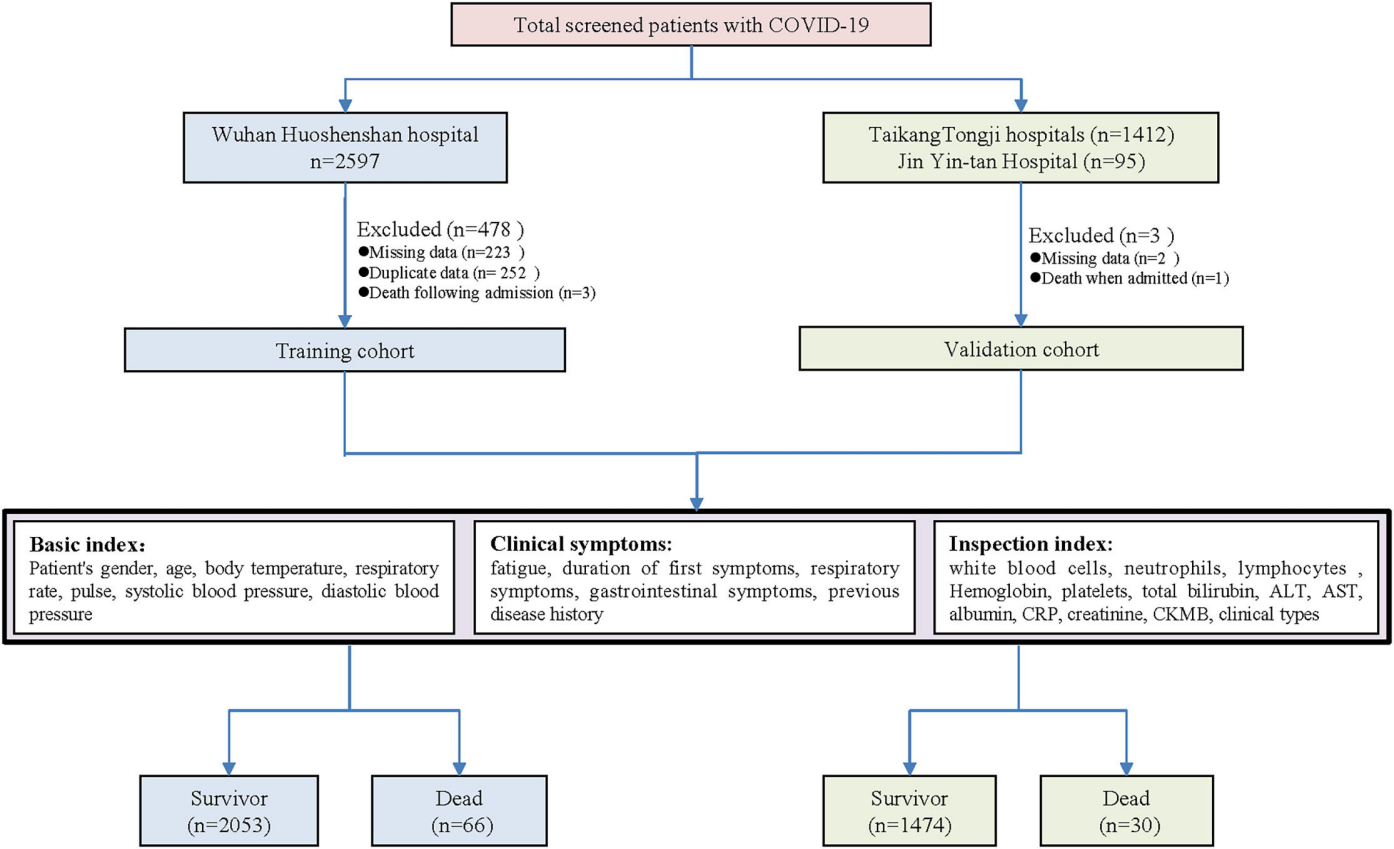
Enrollment flowchart of the study population in the training and validation cohorts.

The eligible patients were enrolled and categorized into the two groups according to the outcome of in-hospital death. The enrollment flowchart is shown in Figure 1. The electronic medical records, nursing records, and laboratory tests of included patients were reviewed by a team consisting of experienced clinicians and statisticians. The dates of admission, discharge, and death were recorded and cross-checked. We collected data on age, sex, pre-existing comorbidities (hypertension, diabetes, cardiovascular diseases, chronic lung diseases, and liver diseases), symptoms from onset to hospital admission (fever, cough, sputum, dyspnea, chest tightness, hemoptysis, fatigue, nausea, abdominalgia, diarrhea, and anorexia), the duration time for initial symptoms, vital signs at hospital admission [body temperature, breathing rate, heart rate, blood pressure [the worst of the first 24 h]], and the basic laboratory values on admission [white blood cell [WBC], neutrophil count, lymphocyte count, hemoglobin [Hg], platelet count [PLT], total bilirubin [TB], alanine aminotransferase [ALT], aspartate aminotransferase [AST], albumin [ALB], C-reactive protein [CRP], creatinine, creatine phosphokinase isoenzyme [CK-MB], interleukin 6 [IL-6], procalcitonin [PCT], and erythrocyte sedimentation rate [ESR]]. The above laboratory tests were carried out in the approved laboratories. The MuLBSTA score for each subject was calculated by the two investigators (EL and JH) as reported previously (8). When disagreement occurred, a senior investigator (SY) decided the final result.

### Statistical Analysis

The variables with missing data over 20% (IL-6, ERS, PCT, etc.) were not included for further statistical analysis. The remaining items were the routine clinical indexes, and the proportion of observation with missing data was <12%. We employed mean substitution for imputation and completed some of the missing data by follow-up with a phone call. The detailed information about missing data was reported in Supplementary Table 1 in the supplement. The continuous variables were expressed as the median [interquartile range (IQR)] or mean ± SD, and categorical variables were presented as *n* (%). The differences in the demographical, clinical characteristics, and laboratory values between the survivors and non-survivors were compared using Mann–Whitney *U*-test, *t*-test, chi-squared test, or Fisher's exact test as appropriate. The predictors with *P*-values <0.05 were fed to a forward stepwise multivariate logistic regression model to identify the independent candidate variables associated with COVID-19 fatality. The factors finally included constructing the nomograms were determined by both the statistical significance and clinical values. To assess the discrimination of established nomograms, the area under the receiver operating characteristic curve (AUC) was measured. A calibration curve was generated for the evaluation of calibration and judged with Hosmer–Lemeshow test (15). The clinical usefulness of the established score was evaluated with the decision curve analysis (DCA) by assessing the net benefits at various threshold probabilities (16). In addition, the performance of the MuLBSTA score was compared with the established nomograms using the same methods described above, and the optimal cut-off value for the MuLBSTA score was adjusted according to Youden's index. The statistical analyses were performed using the IBM SPSS 23 statistics software (SPSS Inc., Chicago, IL, USA) and R software (version 3.4, R Foundation, Vienna, Austria. www.R-project.Org). All the *P*-values were two-sided, and *P* < 0.05 was considered statistically significant.

## Results

### Characteristics of the Training and Validation Cohorts

In total, 2,271 COVID-19 cases were screened from the HSS Hospital, among which 2,119 cases were included as a training set according to the inclusion and exclusion criteria. Total 92 COVID-19 cases from Jin Yin-tan hospital and 1,412 cases from Taikang Tongji hospital were included as a validation cohort (Figure 1). The characteristics of the training and validation cohort were listed in the supplement (Supplementary Table 2). In comparing the training and validation cohort, the majorities of symptoms and part of underlying diseases were similar; whereas some of the laboratory tests were different. The in-hospital mortality rate was 3.1% in the training cohort and 2.0% in the validation cohort.

### Building Nomogram Prognostic Models

**Figure 2:**
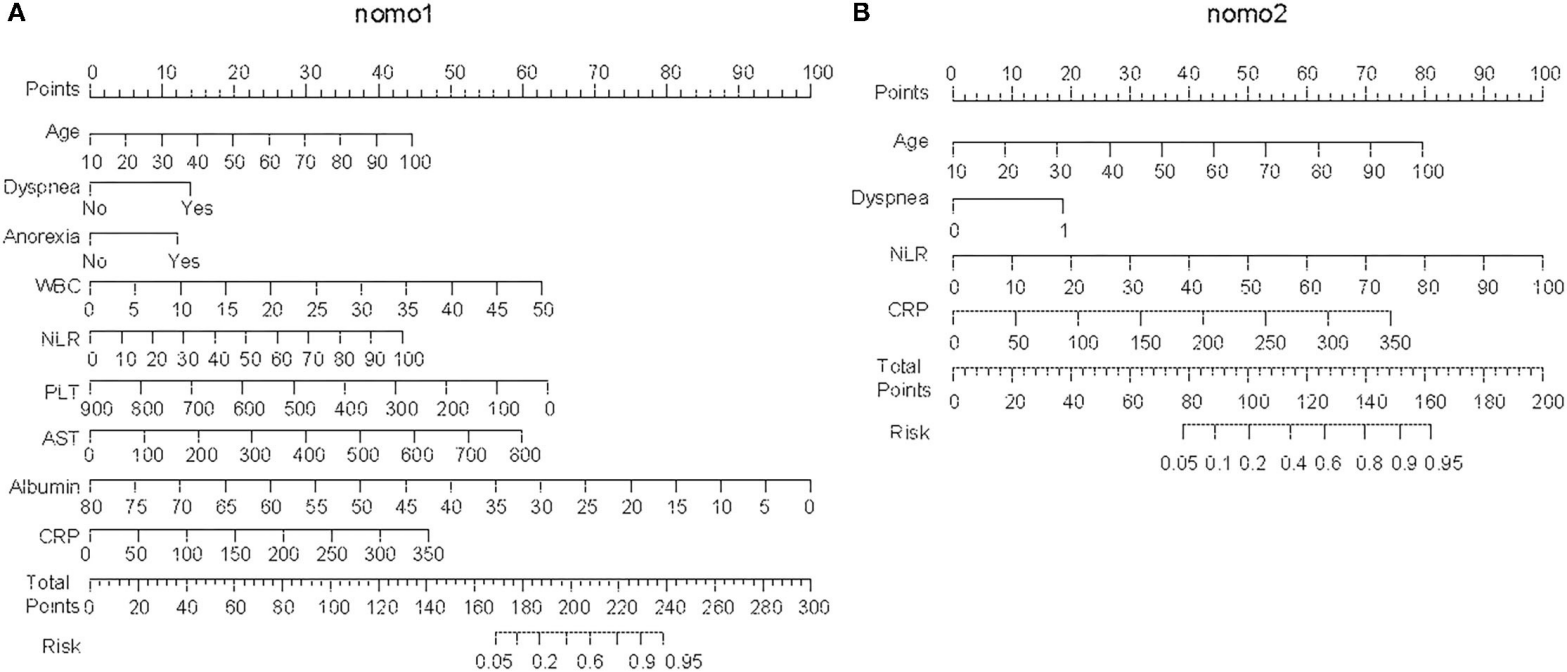
Establishing the nomograms with the identified risk factors. **(A)** Nomo1 was established by including nine identified independent risk factors. **(B)** Nomo2 was established by including age, dyspnea, neutrophil-to-lymphocyte ratio (NLR), and C-reactive protein (CRP).

In univariate analysis, the variables between the survivors and non-survivors in the training cohort were compared, and the *P*-value <0.05 was chosen as the potential factors associated with in-hospital death of COVID-19 (Table 1). The variables chosen above were put in a forward stepwise multivariate logistic regression analysis to explore the independent risk factors. We identified age, dyspnea, anorexia, NLR, PLT, AST, ALB, and CRP as independent risk factors associated with in-hospital mortality of COVID-19 (Table 2). We first built up a full model (Nomo1) with all the indicators above. Considering the clinical practicability of applying a model including nine variables is cumbersome, we then constructed a reduced model (Nomo2) regarding the weight value, the previous reports of significance as well as accessibility in a clinic setting. The nomograms can be used to generate an individual probability of in-hospital death, to fulfill our needs of quick stratifying COVID-19 at the risk of death. To use the nomograms, a ruler ranging from 0 to 100 points was scaled on top, with independent prognostic factors array on the relevant axis below. First, age of a subject was converted to a score by drawing a straight line upward to the ruler on the top and get the score related to age, the procedure was carried out for every covariate, and the final risk score was calculated by adding up the score of each item to estimate the probability of in-hospital death referring to the risk axis at the bottom (Figure 2 and Supplementary Figure 1).

**Table 1:** The demographics, clinical characteristics, vital signs, laboratory findings, and MuLBSTA score between the survivors and non-survivors with COVID-19 [*n* (%)/median (25–75%)/mean ± SD].

	**All (*N* = 2,119)**	**Survivors (*n* = 2,053)**	**Death (*n* = 66)**	***P*** **-value**
Male	1,083 (51.1)	1,041 (50.7)	42 (63.6)	0.039
Age	61.0 (50.0~68.0)	60.0 (50.0~68.0)	69.5 (62.0~78.0)	0.000
Fever	1,496 (70.6)	1,455 (70.9)	41 (62.1)	0.125
Fatigue	1,174 (55.4)	1,129 (55.0)	45 (68.2)	0.034
Respiratory symptoms	1,683 (79.4)	1,625 (79.2)	58 (87.9)	0.084
Cough	1,488 (70.2)	1,440 (70.1)	48 (72.7)	0.651
Sputum	237 (11.2)	233 (11.3)	4 (6.1)	0.180
Dyspnea	651 (29.0)	573 (27.9)	42 (63.6)	0.000
Chest tightness	415 (19.6)	391 (19.3)	18 (27.3)	0.110
Hemoptysis	7 (0.3)	6 (0.3)	1 (1.5)	0.199
Gastrointestinal symptoms	684 (32.3)	652 (31.8)	32 (48.5)	0.004
Vomit	46 (2.2)	43 (2.1)	3 (4.5)	0.171
Abdominal pain	18 (0.8)	17 (0.8)	1 (1.5)	0.436
Diarrhea	100 (4.7)	97 (4.7)	3 (4.5)	1.000
Anorexia	584 (27.6)	553 (26.9)	31 (47.0)	0.000
Duration for initial symptom lasting	20 (13.0~30.0)	20 (14.0~30.0)	14.5 (10.0~25.3)	0.003
Hypertension	678 (32.0)	651 (31.7)	27 (40.9)	0.115
Diabetes	280 (13.2)	263 (12.8)	17 (25.8)	0.002
Cardiovascular disease	122 (5.8)	113 (5.5)	9 (13.6)	0.005
Chronic lung diseases^*^	106 (5.0)	95 (4.6)	11 (16.7)	0.000
Liver diseases^†^	64 (3.0)	62 (3.0)	2 (3.0)	1.000
Body temperature (Mean ± SD)	37.8 ± 1.04	37.8 ± 1.04	37.8 ± 1.14	0.831
Respiratory rate, breaths per min	20.0 (19.0~22.0)	20.0 (19.0~21.0)	22.0 (20.0~26.0)	0.000
Heart rate, beats per min	85.0 (78.0~95.0)	84.0 (78.0~95.0)	88.5 (80.8~101.0)	0.003
SBP, mmHg	129.0 (120.0~140.0)	129.0 (120.0~140.0)	131 (119.8~149.0)	0.250
DBP, mmHg	80.0 (73.0~88.0)	80.0 (74.0~88.0)	80.0 (68.0~88.0)	0.182
WBC, ×109/L	5.7 (4.8~7.1)	5.7 (4.8~7.0)	7.6 (5.6~12.9)	0.000
Neutrophil count, ×109/L	3.5 (2.8~4.7)	3.5 (2.8~4.6)	6.3 (3.7~11.4)	0.000
Lymphocyte count, ×109/L	1.5 (1.1~1.9)	1.5 (1.1~1.9)	0.8 (0.5~1.3)	0.000
NLR	2.4 (1.7~3.5)	2.4 (1.7~3.4)	11.1 (2.8~21.6)	0.000
Hemoglobin concentration, g/L	124.0 (113.0~135.0)	124.0 (113.8~135.0)	115 (102.3~131.0)	0.001
Platelet count, ×109/L	226.0 (183.0~279.0)	227.0 (185.0~279.0)	191.0 (88.5~265.3)	0.000
Total bilirubin concentration, μmol/L	9.5 (7.3~12.3)	9.4 (7.3~12.2)	10.9 (8.3~18.5)	0.000
ALT, IU/L	24.1 (15.0~38.9)	24.1 (15.0~38.8)	25.7 (15.9~42.9)	0.486
AST, IU/L	19.9 (15.7~27.1)	19.8 (15.7~26.6)	27.5 (17.5~47.2)	0.000
Albumin concentration, g/L	37.5 (34.6~40.1)	37.6 (34.7~40.2)	31.8 (28.0~35.1)	0.000
CRP, mg/L	2.4 (0.9~9.8)	2.3 (0.9~8.7)	53.2 (6.5~140.5)	0.000
Serum creatinine concentration, μmol/L	64.5 (55.0~75.7)	64.3 (55.0~75.3)	68.7 (55.9~87.8)	0.021
CK-MB concentration, IU/L	9.1 (7.0~13.6)	9.0 (7.0~13.3)	113.9 (8.7~22.7)	0.000
MuLBSTA score	7.0 (5.0~9.0)	7.0 (5.0~9.0)	11.0 (9.0~13.5)	0.000

*WBC, White blood cells; NLR, Neutrophil to lymphocyte ratio; ALT, alanine aminotransferase; AST, aspartate aminotransferase; CRP, C reactive protein; CK-MB, Creatine kinase-MB.*

* *Including bronchitis, chronic obstructive pulmonary diseases, pulmonary tuberculosis, and lung tumors.*

† *Including Hepatitis (A, B, C, D, and E), cirrhosis, fatty liver, and liver tumors*.

**Table 2:** The multivariate logistic regression modes used to construct Nomo1 and Nomo2 (*N* = 2,119).

		**OR**	**OR 95% CI**		***P*** **-value**
			**Lower limit**	**Upper limit**	
Nomo1	Age	1.043	1.017	1.071	0.001
	Dyspnea	3.306	1.806	6.052	0.000
	Anorexia	2.828	1.535	5.208	0.001
	WBC	1.114	1.040	1.192	0.002
	NLR	1.038	1.005	1.072	0.023
	PLT	0.994	0.990	0.998	0.001
	AST	1.006	1.002	1.011	0.004
	Albumin	0.898	0.843	0.958	0.001
	CRP	1.012	1.006	1.017	0.000
Nomo2	Age	1.064	1.038	1.091	0.000
	Dyspnea	3.682	2.066	6.564	0.000
	NLR	1.073	1.039	1.107	0.000
	CRP	1.015	1.010	1.020	0.000

### The Performance of Nomograms and the Comparison With MuLBSTA Score in the Training Cohort

**Figure 3:**
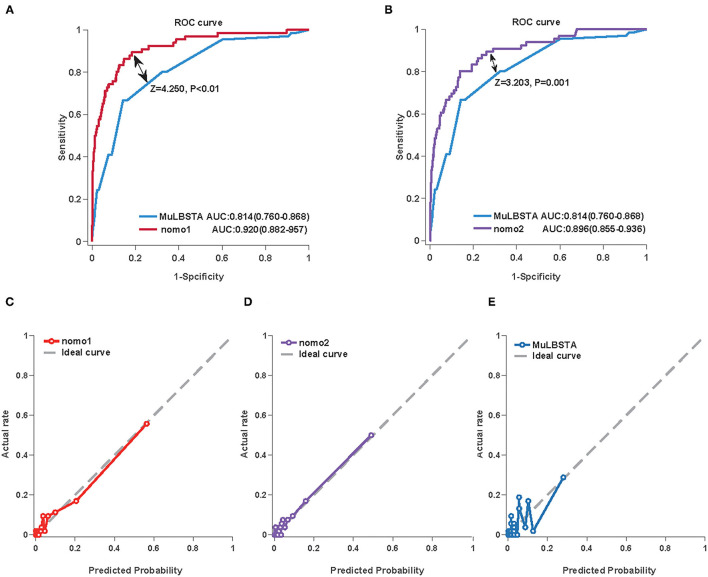
The performance of the Nomo1, Nomo2, and the MuLBSTA score in predicting the mortality of COVID-19 in the training cohort. The area under the curve (AUC) curve analyses were generated to test the discrimination of Nomo1 and the MuLBSTA score **(A)** or Nomo2 and the MuLBSTA score **(B)**. The calibration curves were generated for Nomo1 **(C)**, Nomo2 **(D)**, and the MuLBSTA score **(E)**. The dashed line represents an ideal prediction.

We first used AUC to compare the discrimination of the established nomograms and MuLBSTA score in predicting the mortality of COVID-19. Both Nomo1 and Nomo2 performed better discrimination than MuLBSTA score (AUC_Nomo1_ = 0.920, 95% *CI* 0.882–0.957 vs., AUC_MuLBSTA_ = 0.814, 95% *CI* 0.76–0.868, *P* < 0.001; AUC_Nomo2_ = 0.896, 95% *CI* 0.855–0.936 vs. AUC_MuLBSTA_ = 0.814, 95% *CI* 0.76–0.868, *P* = 0.001) (Figures 3A,B and Supplementary Table 3). The calibration curves of Nomo1 and Nomo2 showed high consistency between predicted survival probability and actual survival proportion, better than the calibration curve of MuLBSTA score (Figures 3C–E). Furthermore, the generated curve of DCA indicated that employing the Nomo1 and Nomo2 to identify the patients with a high risk of death would be advantageous over the MuLBSTA score (Supplementary Figure 2).

### External Validation of the Nomograms

**Figure 4:**
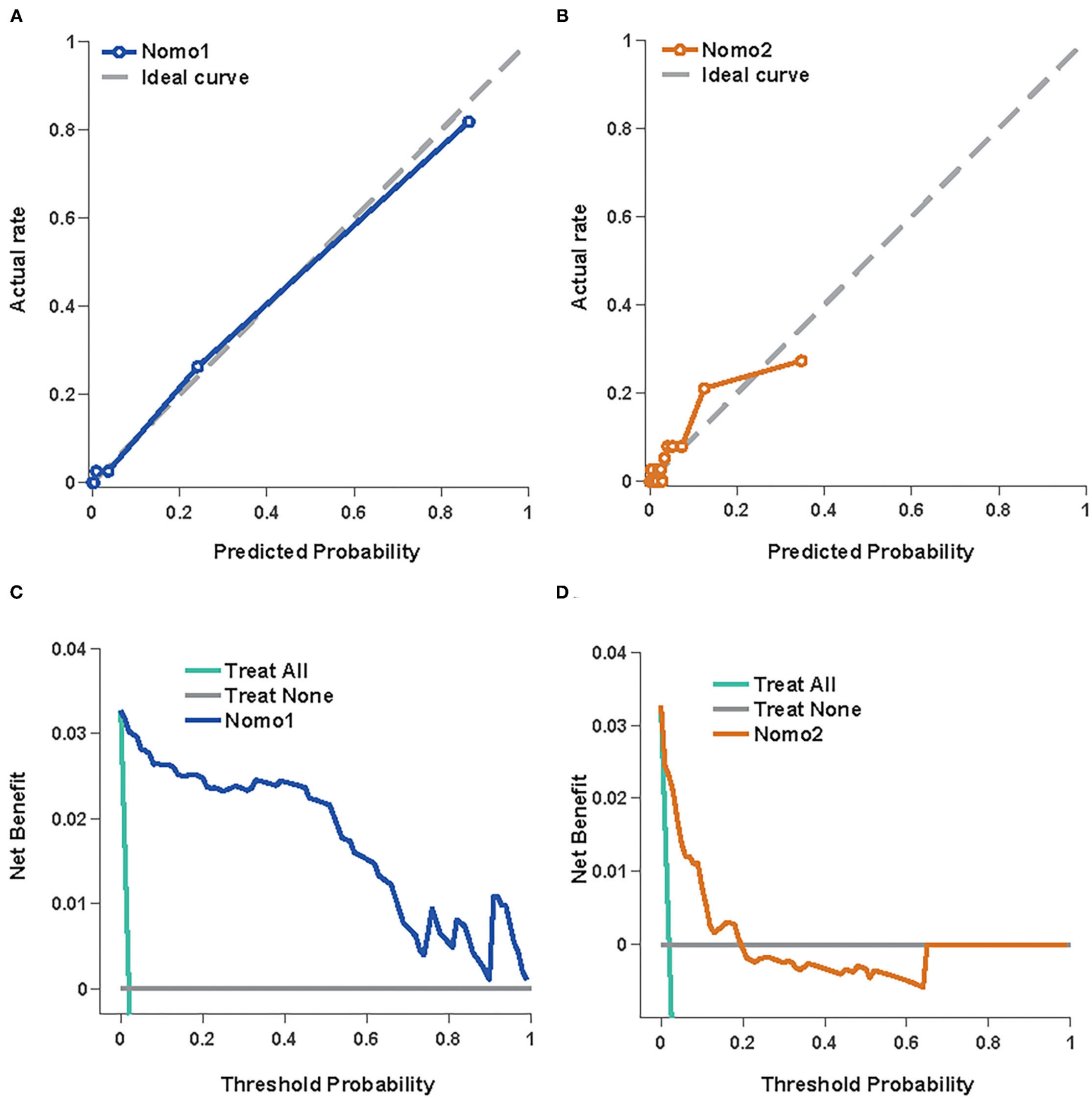
Nomo1and Nomo2 in predicting the mortality of COVID-19 in the validation cohort. The calibration curves for Nomo1 **(A)** and Nomo2 **(B)**. A decision curve analysis for Nomo1 **(C)** and Nomo2 **(D)**.

The predictive values of Nomo1 and Nomo2 were further validated in the external datasets. The Nomo1 showed an AUC of 0.92 (95% *CI* 0.86–0.98) and Nomo2 showed an AUC of 0.89 (95% *CI* 0.83–0.96) in the validation cohort (Figures 4A,B and Table 3). For calibration, Nomo1 showed a better agreement of observed proportion of death with predicted one than Nomo2 presented. Overall, the DCA also showed that Nomo1 is more beneficial in predicting the mortality of the patients with COVID-19 than Nomo2 in a validation cohort (Figures 4C,D).

**Table 3:** The nomograms in predicting the COVID-19 mortality with external datasets (*N* = 1,504).

	***N***	**AUC** **(95 %CI)**	**Sen** **(%, 95%CI)**	**Spe** **(%, 95%CI)**	**Acc** ***n*** **(%, 95%CI)**	**Youden's index**
Nomo1	1,504	0.92 (0.86–0.98)	86.67 (74.50–98.83)	88.74 (87.12–90.35)	88.70 (87.10–90.30)	0.75
Nomo2	1,504	0.89 (0.83–0.96)	76.67 (61.53–91.80)	89.82 (88.28–91.37)	89.56 (88.02–91.11)	0.66

*AUC, Area under the curve; Sen, Sensitivity; Spe, specificity; Acc, accuracy*.

## Discussion

Coronavirus disease 2019 pandemic has emerged as one of the greatest challenges of our time and has caused more the 4 million deaths up to now. To control the mortality of COVID-19 is of greatest importance to reduce the public crisis and health budget. Considering no effective anti-viral medicine and breakthrough infections after vaccination, how to recognize the patients who are at risk of death became an urgent issue. In consideration of those current predictive models that were built by a relatively small number of patients, and some indicators included were not routinely detected in the out-patient department, emergency room, or underdeveloped regions or countries, we explored the routine clinical predictive factors and developed nomograms to predict the risk of death for the patients with COVID-19 using a total of 3,623 cases.

In this study, we have identified nine factors, including age, dyspnea, anorexia, WBC, NLR, PLT, AST, ALB, and CRP, that were the independent risk factors for COVID-19 fatality. Age is an acknowledged risk factor associated with disease severity and prognosis of COVID-19 (17). The mortality increased sharply to 7.8% in the aged patients over 80, while the overall death rate was estimated to be 0.66% (18). In our study, the median ages in the survivors and non-survivors were 60.0 (49.3~68.0) and 69.0 (62.0~78.0). Zhou et al. reported the median age of non-survivors was 69 (63.0–76.0) in China (19). A study carried out in Italy, showed 69 years or older had significantly decreased survival probability compared with the younger patients [HR: 4.25 (3.68–4.92)] (20). Another study in New York showed the median age of non-survivors was 68 (60.0–75.0) (21).

Dyspnea can reflect the severity of the disease as it was associated with an increased risk of developing acute respiratory distress syndrome (ARDS) in another study (3). We observed that 18 (25.35%) non-survivors were common type when admitted, who reported symptoms of dyspnea but no respiratory distress, ahead of their subsequent disease progression into severe type. The involvement of dyspnea in the score compensates for underestimating death in patients in the early stage before disease progression. Another symptom, anorexia, is an independent predictor of death in our study. Consistently, gastrointestinal involvement has been observed in the patients with COVID-19 (22, 23) and anorexia was associated with intensive care unit (ICU) admission for the patients with COVID-19 (24).

For laboratory parameters, a higher neutrophil count and lymphocytopenia were noted in the non-survivors. Lymphocytopenia is a characteristic of severe patients with COVID-19 since the lymphocyte count in the ICU patients was 0.4 (0.2–0.8) compared with 1.0 (0.7–1.1) in non-ICU patients (25). The lymphocyte count was integrated into a predictive model for COVID-19 fatality (26). The net effect of elevated neutrophils and decreased lymphocytes resulted in raised NLR. The value of NLR ≥ 2.22 had been used to recognize COVID-19, and NLR ≥ 4.06 was an indicator of severe disease (27).

In our study, the PLT count was lower in non-survivors compared with the survivors [190 (87~265) vs. 227 (184~280), *p* < 0.01], which is in line with a previous report (7). However, PLT was reported to be significantly higher in the ICU patients than in the non-ICU patients (25), while the study carried out in Jin Yin-tan Hospital that enrolled 52 critically ill patients showed that the non-survivors had elevated PLT (28). We speculate that the difference between these studies might be influenced by selection bias and the number of patients enrolled.

The CRP, significantly elevated in the non-survivors compared with the survivors [52.38 (7.76~132.7) vs. 2.46 (0.92~10.07) mg/L], was an independent risk factor for mortality of COVID-19. Similar to our study, a retrospective study delineating the clinical characteristics of 113 non-survivors with COVID-19 showed that the CRP was significantly elevated in deaths compared with recovered patients with severe diseases [113 (69.1–168.4) vs. 26.2 (8.7–55.8)] (29).

Regarding the liver function, AST and TB were higher while ALB was lower in the non-survivors compared with the survivors, while ALT was comparable between these two groups. A meta-analysis including 35 studies of 6,686 patients with COVID-19 showed that the pooled prevalence of abnormal liver function was 19%. ALT, AST, and TB predicted severe cases with pooled odds ratios (*OR*s) of 1.89 (1.30–2.76), 3.08 (2.14–4.42), and 1.39 (0.78–2.47), respectively (3).

Here, based on these predictive factors, the two nomograms to evaluate the mortality risk of COVID-19 were developed, validated, and compared with the MulBSTA score. The MuLBSTA score was previously used to predict the mortality risk of viral pneumonia. The model was established mainly by the patients with influenza pneumonia and other viral pneumonia. Seven parameters, including multilobular infiltrates, lymphocytes, bacterial coinfection, acute smoker, former smoker, hypertension, and age ≥60 years were included. It has been reported that the death with COVID-19 had high MuLBSTA scores (2). In our study, the non-survivors had higher MuLBSTA scores than the survivors [11 (7~13) vs. 7 (5~9), *P* < 0.001]. However, the AUC of the MuLBSTA score was 0.814 [95% *CI* 0.76–0.868], with a sensitivity of 40.91% (28.79–53.03%). The poor sensitivity of the MuLBSTA score made it unsuitable for predicting the mortality risk of COVID-19. By adjusting the optimal cut-off value from the reported 12–10.5 according to the Youden Index, the sensitivity of the MuLBSTA score increased to 66.67% (95% *CI* 54.55–77.27%).

The medical nomograms, using biological and clinical variables to determine a probability of a clinical event, are a pictorial representation of a predictive model. In the present study, we constructed two nomograms, Nomo1 and Nomo2, that predicted the mortality of COVID-19 with AUC_nomo1_ of 0.92 (95% *CI* 0.86–0.98) and AUC_nomo2_ of 0.89 (95% *CI* 0.83–0.96) in validation. Nomo1 consisted of nine indexes, the procedure to calculate the total score by adding the nine scores together is slightly cumbersome and time-consuming. To get a more user-friendly score, we constructed a reduced model Nomo2 regarding the weight value, the previous reports of significance as well as accessibility in a clinic setting. Age, dyspnea, NLR, and CRP were included to construct Nomo2 and showed AUC_nomo2_ of 0.89 (95% *CI* 0.83–0.96) in validation. In an out-patient or emergency department, the risk of COVID-19 mortality can be acquired easily using Nomo2 while we recommended the use of Nomo1 in general hospitals.

A number of similar studies before us have constructed nomograms to predict the outcome of COVID-19. Nguyen constructed a nomogram using 279 hospitalized patients with COVID-19 to predict the 14-day probability of an unfavorable outcome defined as the need for artificial ventilation and/or death. Age, male gender, BMI, respiratory rate, body temperature, lymphocyte count, CRP, and TNI were used to construct the predictive nomogram with a C-statistics of 0.75 (10). However, it was a single-center study without external validation of the model, and the definition of endpoint consisted of the patients receiving artificial ventilation. Furthermore, the factors, such as TNI (10, 11), D-dimer (12), NT-proBNP (13) included were not routine laboratory tests, and the application of the nomograms was hindered in out-patient department, mobile cabin hospitals, or emergency healthcare centers. Compared with the above nomograms, our models included a large number of patients with COVID-19; all the variables included in the nomogram are routine clinical indexes, making it applicable in most medical institutions worldwide; the two nomograms were built for different purposes, Nomo1 is more robust and Nomo2 is more convenient, a clinician may choose either one appropriate according to the situation.

As with any retrospective study, there are several limitations in our study. First, as a retrospective study, bias is inevitable, and the results should be interpreted carefully as an exploratory study. Second, since the study was carried out in a single city (Wuhan, China), the results are not fully representative. Third, from the beginning of 2021, the new virus strains, such as B.1.617.2 (Delta) variant spread worldwide, showed increased ability to transmit, pathogenicity, and lethality, the predictive potency of the nomogram needs to be verified in countries and areas with new virus strains. However, despite these limitations, we have successfully identified the in-hospital mortality risk factors of COVID-19 and have constructed predictive nomograms to estimate the in-hospital mortality risk of COVID-19 based on the routine clinical indicators.

## Data Availability Statement

The original contributions presented in the study are included in the article/Supplementary Material, further inquiries can be directed to the corresponding author/s.

## Ethics Statement

The studies involving human participants were reviewed and approved by the Ethics Committee of Xinqiao Hospital (2020-yd073-01). Written informed consent from the participants' legal guardian/next of kin was not required to participate in this study in accordance with the national legislation and the institutional requirements.

## Author Contributions

SY and YX: had full access to all the data in the study and are responsible for the authenticity, accuracy of the data manipulation, and analysis. SY, MH, and YX: design and organization. JH, XL, YL, XR, CZ, WZ, WD, YT, and PL: data collection, extraction, and cleansing. EL and HL: statistical analysis. JH and YX: drafting of the manuscript. QL and ZX: technical support. HW and CS: administrative support. All authors contributed to the article and approved the submitted version.

## Funding

This work was supported by a Joint grant from the Science/Technology Commission of Chongqing and the National Health Commission of Chongqing, China (Grant Number: 2020FYYX115).

## Conflict of Interest

The authors declare that the research was conducted in the absence of any commercial or financial relationships that could be construed as a potential conflict of interest.

## Publisher's Note

All claims expressed in this article are solely those of the authors and do not necessarily represent those of their affiliated organizations, or those of the publisher, the editors and the reviewers. Any product that may be evaluated in this article, or claim that may be made by its manufacturer, is not guaranteed or endorsed by the publisher.
