# Supplementary material for: Establishment of Routine Clinical Indicators-Based Nomograms for Predicting the Mortality in Patients With COVID-19
Source: Front Med (Lausanne). 2021 Oct 18;8:706380. doi: 10.3389/fmed.2021.706380 (PMC8558233; doi:10.3389/fmed.2021.706380)
Supplement: Supplementary file 1 [file Data_Sheet_1.pdf]

Supplementary Material

1 Supplementary Figures and Tables

1.1 Supplementary Figures

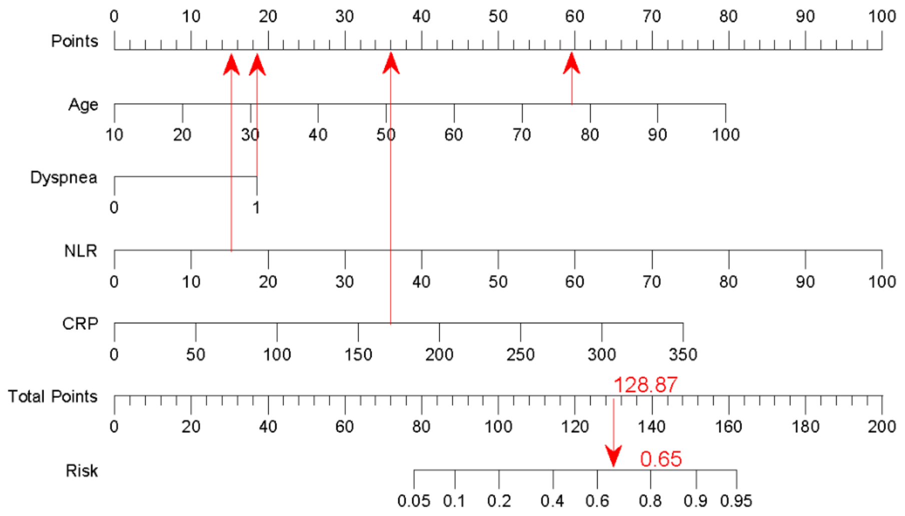

sFigure 1. Example of adopting nomo2 in the assessment of in-hospital mortality probability.

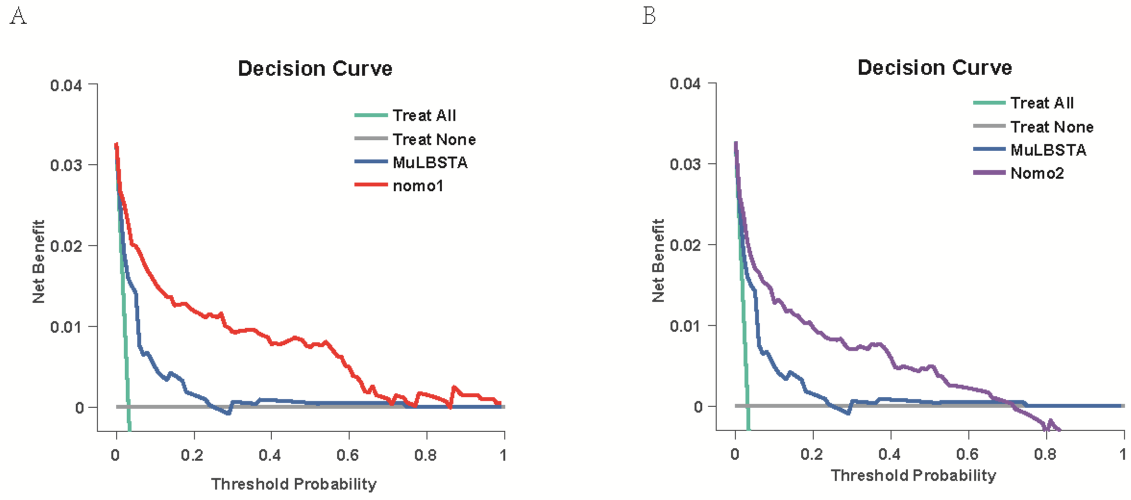

sFigure2. Decision curve analysis of nomo1, nomo2 and the MulBSTA score. (A)Comparison

between nomo1 and the MulBSTA score. The blue line represents the DCA of the MulBSTA score, and the red line represents the DCA of the nomo1. (B) Comparison between nomo2 and the MulBSTA score. The blue line represents the DCA of the MulBSTA score, and the purple line represents the DCA of the nomo2.

1.2 Supplementary Tables

sTable 1 Percentage of missing data for variables of patients from Huo-shen-shan hospital (N=2119)

variables	missing (n)	%	treatment	Before imputation	After imputation
Sex	0	0.00	..	..	..
Age	0	0.00	..	..	..
Fatigue	0	0.00	..	..	..
Cough	0	0.00	..	..	..
Sputum	0	0.00	..	..	..
Dyspnea	0	0.00	..	..	..
Chest tightness	0	0.00	..	..	..
Hemoptysis	0	0.00	..	..	..
Vomit	0	0.00	..	..	..
Abdominal pain	0	0.00	..	..	..
Diarrhea	0	0.00	..	..	..
Anorexia	0	0.00	..	..	..
MuLBSTA score	0	0.00	..	..	..
death	0	0.00	..	..	..

Severe/Critical type	0	0.00	..	..	..
Hypertension	0	0.00	..	..	..
Diabetes	0	0.00	..	..	..
Cardiovascular disease	0	0.00	..	..	..
Chronic lung diseases	0	0.00	..	..	..
Liver diseases	0	0.00	..	..	..
Heart rate	7	0.33	mean substitution	84.5(78~95）	85(78~95）
Respiratory rate	9	0.42	mean substitution	20(19~22）	20(19~22）
Body temperature（mean±SD）	11	0.52	mean substitution	37.74±1.04	37.74±1.04
SBP, mmHg	11	0.52	mean substitution	129(120~140）	129(120~140）
DBP, mmHg	13	0.61	mean substitution	80(73~88）	80(73~88）
Duration for initial symptom lasting	31	1.46	phone call	20(13~30）	20(13~30）
WBC	109	5.14	mean substitution	5.7(4.8~7.1）	5.7(4.8~7.1）
Lymphocyte count	109	5.14	mean substitution	1.5(1.09~1.86）	1.49(1.1~1.85）
NLR	109	5.14	mean substitution	2.35 (1.69~3.46）	2.38(1.72~3.53）

Hemoglobin concentration	118	5.57	mean substitution	124(113~135）	124(113~135）
Neutrophil count	120	5.66	mean substitution	3.51(2.74~4.69）	3.53(2.76~4.7）
Platelet count	128	6.04	mean substitution	225(182~279）	226(183~279）
Albumin concentration	131	6.18	mean substitution	37.6(34.6~40.2）	37.5(34.6~40.1）
ALT	139	6.56	mean substitution	23.4(14.8~38.4）	24.1(15~38.9）
AST	149	7.03	mean substitution	19.6(15.6~26.63）	19.9(15.7~27.1）
Total bilirubin	157	7.41	mean substitution	9.4(7.2~12.3）	9.45(7.3~12.3）
CRP	170	8.02	mean substitution	2.25(0.85~8.74）	2.42(0.91~9.77）
Serum Cr concentration	180	8.49	mean substitution	64.1(54.6~75.9）	64.5(55~75.7）
CKMB	245	11.56	mean substitution	9(6.9~13.4）	9.1(7~13.6）
D-dimer	468	22.09	excluded	..	..
PCT	858	40.49	excluded	..	..
Anti-SARS-COV2-IgG	1061	50.07	excluded	..	..
Anti-SARS-COV2-IgM	1066	50.31	excluded	..	..

IL-6	1323	62.4<br>4	excluded	..	..
Myoglobin	1355	63.9<br>5	excluded	..	..
ESR	1993	94.0<br>5	excluded	..	..

Abbreviations:WBC White blood cells, NLR Neutrophil to lymphocyte ratio, ALT alanine aminotransferase, ASTaspartate aminotransferase, CRP C reactive protein, CK-MB Creatine kinase-MB， Cr creatinine, IL-6 interleukin 6, ESR erythrocyte sedimentation rate.

sTable 2 Demographics and clinical characteristics of COVID-19 patients [n(%)/Median 25%-75%)]

	**Training cohort (N=2119)**	**Validation cohort (N=1504)**	**P value**
Male [n(%)]	1083(51.1)	683 (45.4)	0.001
Age	61.0(50.0~68.0)	62.0(51.0~70.0)	0.000
Fever [n(%)]	1496(70.6)	823 (54.7)	0.000
Fatigue [n(%)]	1174(55.4)	625 (41.6)	0.000
Respiratory symptoms [n (%)]	1683(79.4)	682 (45.3)	0.000
Cough	1488(70.2)	863 (57.4)	0.000
Sputum	237(11.2)	211 (14.0)	0.010
Dyspnea	615 (29.0)	262 (17.4)	0.000
Chest tightness	415(19.6)	318 (21.1)	0.250
Hemoptysis	7(0.3)	1(0.1)	0.151
Gastrointestinal symptoms [n(%)]	684(32.3)	537 (35.7)	0.032
Vomit	46(2.2)	38 (2.5)	0.483
Abdominal pain	18(0.8)	24(1.6)	0.039
Diarrhea	100(4.7)	86 (5.7)	0.179
Anorexia	584(27.6)	371 (24.7)	0.052
Duration for initial symptom lasting	20(13.0~30.0)	22.5 (14.0~30.0)	0.162
Hypertension [n (%)]	678(32.0)	225 (15.0)	0.000
Diabetes [n (%)]	280(13.2)	45(3.0)	0.000
Cardiovascular disease [n (%)]	122(5.8)	12(0.8)	0.000
Chronic lung diseases [n (%)]	106(5.0)	53(3.5)	0.032
Liver diseases [n (%)]	64(3.0)	43(2.9)	0.778

Clinical classfication [n (%)]			0.044
Common type	1568(74.0)	1157 (76.9)	
Severe/Critical type	551(26.0)	347 (23.1)	
Body temperature (Mean±SD)	37.8±1.0	37.4±1.0	0.000
Respiratory rate, breaths per min	20.0 (19.0~22.0	20.0 (18.0~21.0)	0.000
Heart rate, beats per min	85.0 (78.0~95.0)	84.0 (78.0~96.0)	0.872
SBP, mmHg	129.0(120.0~140.0)	130.0 (120.0~1141.0)	0.067
DBP, mmHg	80.0(73.0~88.0)	80 (72.0~88.0)	0.760
WBC, $\times 10^9$/L	5.7(4.8~7.1)	5.8 (4.8~6.8)	0.556
Neutrophil count, $\times 10^9$/L	3.5(2.8~4.7)	3.4 (2.6~4.3)	0.000
Lymphocyte count, $\times 10^9$/L	1.5(1.1~1.9)	1.6(1.2~1.9)	0.001
NLR	2.4(1.7~3.5)	2.2 (1.5~3.1)	0.000
Hemoglobin concentration, g/L	124.0(113.0~135.0)	119.0(109.0~130.0)	0.000
Platelet count, $\times 10^9$/L	226.0(183.0~279.0)	225.0(182.3~263.0)	0.009
Total bilirubin concentration, μmol/L	9.5(7.3~12.3)	11.4(8.8~13.4)	0.000
ALT, IU/L	24.1(15.0~38.9)	24.6(15.1~36.3)	0.982
AST, IU/L	19.9(15.7~27.1)	36.1(19.7~37.4)	0.000
Albumin concentration, g/L	37.5(34.6~40.1)	37.8(35.2~40.5)	0.001
CRP,mg/L	2.4(0.9~9.8)	0.8 (0.5~6.4)	0.000
Serum Cr concentration, μmol/L	64.5(55.0~75.7)	58.4(46.9~75.9	0.000
CK-MB, IU/L	9.1(7.0~13.6)	8.6(6.2~10.0)	0.000
In-hospital death	66(3.1)	30(2.0)	0.039

Abbreviations: WBC White blood cells, NLR Neutrophil to lymphocyte ratio, ALT alanine aminotransferase, ASTaspartate aminotransferase, CRP C reactive protein, CK-MB Creatine kinase-MB，Cr creatinine

sTable3 Nomo1, Nomo2 and MuLBSTA score in predicting mortality of COVID-19 in training cohort （N=2119）

	Sen (%,95 CI)	Spe (%,95 CI)	+LR	-LR	Acc (%,95 CI)	Youden index
Nomo1	86.36 (77.27-93.94)	85.24 (83.63-88.95)	5.85	0.16	85.28 (83.81-89.15)	0.72
Nomo2	80.30 (70.71-89.90)	86.12 (84.62-87.61)	5.78	0.22	85.94 (84.46-87.42)	0.66
MuLBSTA score*	40.91 (28.79-53.03)	90.98 (89.44-92.11)	4.54	0.64	89.43 (87.83-90.56)	0.32
MuLBSTA score#	66.67 (54.55-77.27)	85.78 (84.07-87.25)	4.68	0.39	85.18 (83.39-86.50)	0.52

Sen=sensitivity. Spe=specificity. +LR=positive likelihood ratio. -LR=negative likelihood ratio. Acc=accuracy. * cut-off value=12. # cut-off value=10.5.

sTable 4 Nomograms for predicting COVID-19 mortality with external datasets. (N=1504)

	N	AUC (95% CI)	Sen (%, 95% CI)	Spe (%, 95% CI)	Acc n(%, 95% CI)	Youden's index
Nomo1	1504	0.92 (0.86-0.98)	86.67 (74.50-98.83)	88.74 (87.12-90.35)	88.70 (87.10-90.30)	0.75
Nomo2	1504	0.89 (0.83-0.96)	76.67 (61.53-91.80)	89.82 (88.28-91.37)	89.56 (88.02-91.11)	0.66

Note: AUC=Area under the curve. Sen=Sensitivity. Spe =specificity. Acc= accuracy
