# Supplementary material for: Establishment of Routine Clinical Indicators-Based Nomograms for Predicting the Mortality in Patients With COVID-19
Source: Front Med (Lausanne). 2021 Oct 18;8:706380. doi: 10.3389/fmed.2021.706380 (PMC8558233; doi:10.3389/fmed.2021.706380)
Supplement: Supplementary file 2 [file Data_Sheet_2.DOCX]

| **Section/Topic** | **Item** |  | **Checklist Item** | **Page** |
| --- | --- | --- | --- | --- |
| **Title and abstract** | | | | |
| Title | 1 | D;V | Identify the study as developing and/or validating a multivariable prediction model, the target population, and the outcome to be predicted. | “Establishment of routine clinical indicators-based nomograms for predicting the mortality in patients with COVID-19” |
| Abstract | 2 | D;V | Provide a summary of objectives, study design, setting, participants, sample size, predictors, outcome, statistical analysis, results, and conclusions. | OBJECTIVE/ STUDY DESIGN/ SETTING/ PARTICIPANTS/ OUTCOME:  Objectives: The aim of the study was to establish and validate nomograms to predict the mortality risk of patients with COVID-19 using routine clinical indicators  study design: retrospective study.  participants: Hospitalized COVID-19 patients from Huo-Shen-Shan Hospital, Jin-Yintan Hospital and Taikang Tongji Hospital, Wuhan, China  SAMPLE SIZE: “N total=3623”  PREDICTORS: The demographics, clinical manifestations, vital signs and laboratory test results of the patients at admission and outcome of in-hospital death were recorded  RESULTS: “Nomogram 1 and nomogram 2 established showed better performance in discrimination and calibration than the MuLBSTA score in training. The models were then tested in an external dataset with AUC of 0.91(95% CI 0.85-0.98) for nomogram 1 and 0.93 (95% CI 0.90-0.97) for nomogram 2. Although nomogram 1 performed better than nomogram 2 in calibration, nomogram 2 is more convenience for clinical application.” |
| **Introduction** | | | | |
| Background and objectives | 3a | D;V | Explain the medical context (including whether diagnostic or prognostic) and rationale for developing or validating the multivariable prediction model, including references to existing models. | Introduction:  “The MuLBSTA score [8], developed to assess the outcome of viral pneumonia, was reported to be associated with the outcome of COVID-19 in a few observational studies during the initial outbreak of COVID-19 in Wuhan (China) [9]. COVID-19 differed significantly from influenza A, rhinovirus, and other respiratory virus pneumonias that were used to build up the MuLBSTA score. Moreover, dichotomous classification of mortality risk as low or high risk, again, forced the clinician to make decision himself for the priority of medical resource usage when large number of patients are classified in the same group, which in turn minimize the usefulness of model prediction. Nomogram is a good method for this purpose with visualized interface and consecutive risk prediction. To date there are three studies of nomograms in predicting death risk in COVID-19 [10-11]. The nomograms developed from those studies provided useful tools for researchers and clinicians in stratifying COVID-19 patients. However, the size of participants enrolled were limited in all three studies, and a lack of independent validation was noted in one study [10]. Furthermore, factors such as troponin (TNI), lactate dehydrogenase (LDH) included are not routine laboratory tests, the application of the nomograms are not suitable in mobile cabin hospitals or emergency health care centers” |
|  | 3b | D;V | Specify the objectives, including whether the study describes the development or validation of the model or both. | Introduction: Paragraph 3  Describes both |
| **Methods** | | | | |
| Source of data | 4a | D;V | Describe the study design or source of data (e.g.,randomized trial, cohort, or registry data), separately for the development and validation datasets, if applicable. | Methods: Study design and participants |
|  | 4b | D;V | Specify the key study dates, including start of accrual; end of accrual; and, if applicable, end of follow-up. | Methods: Study design and participants  “Discovery: patients admitted to HSS Hospital from Feb 4, 2020, to Mar 31, 2020, were retrospectively screened and were followed up to April 15, 2020  Methods:Study design and participants  includedCOVID-19 patients admitted to Jin Yin-tan Hospital (Wuhan City, China) from Jan 26, 2020, to Feb 1, 2020” and COVID-19 patients admitted to Taikang Tongji Hospital (Wuhan City, China) from Feb 19, 2020, to Apr 2, 2020. |
| Participants | 5a | D;V | Specify key elements of the study setting (e.g., primary care, secondary care, general population) including number and location of centres. | Methods: Procedure and data collection |
|  | 5b | D;V | Describe eligibility criteria for participants. | Methods: Inclusion and exclusion criteria |
|  | 5c | D;V | Give details of treatments received, if relevant. | N/A (retrospective study, treatments were not included) |
| Outcome | 6a | D;V | Clearly define the outcome that is predicted by the prediction model, including how and when assessed. | Methods: Procedure and data collection  And results  “Eligible patients were enrolled and categorized into two groups according to the outcome of in-hospital death” |
|  | 6b | D;V | Report any actions to blind assessment of the outcome to be predicted. | N/A (retrospective study) |
| Predictors | 7a | D;V | Clearly define all predictors used in developing or validating the multivariable prediction model, including how and when they were measured. | Methods: Procedure and data collection and eMethods in supplementary data |
|  | 7b | D;V | Report any actions to blind assessment of predictors for the outcome and other predictors. | N/A (retrospective study) |
| Sample size | 8 | D;V | Explain how the study size was arrived at. | N/A (retrospective study) |
| Missing data | 9 | D;V | Describe how missing data were handled (e.g., complete-case analysis, single imputation, multiple imputation) with details of any imputation method. | Methods: Variables with missing data over 20% (IL-6, ERS and PCT, etc) were not included for further statistical analysis. Detailed information about missing data are reported in eTable1 in the supplement. The remaining items were actually routine clinical indexes, and the proportion of observation with missing data was less than 12%. We employed mean substitution for imputation and completed some of the missing data by follow-up with a phone call.” |
| Statistical analysis methods | 10a | D | Describe how predictors were handled in the analyses. | Methods: Statistical analysis |
|  | 10b | D | Specify type of model, all model-building procedures (including any predictor selection), and method for internal validation. | Methods: Statistical analysis  And results |
|  | 10c | V | For validation, describe how the predictions were calculated. | Methods: Procedure and data collection |
|  | 10d | D;V | Specify all measures used to assess model performance and, if relevant, to compare multiple models. | Methods:  “Statistical analysis”  Nomogram1, nomogram2 and the MulBASTA score |
|  | 10e | V | Describe any modelupdating (e.g., recalibration) arising from the validation, if done. | N/A |
| Risk groups | 11 | D;V | Provide details on how risk groups were created, if done. | the outcome of in-hospital death was identified by reviewing electronic records of included subjects |
| Development vs. validation | 12 | V | For validation, identify any differences from the development data in setting, eligibility criteria, outcome, and predictors. | supplementary data sTable 2 |
| **Results** | | | | |
| Participants | 13a | D;V | Describe the flow of participants through the study, including the number of participants with and without the outcome and, if applicable, a summary of the follow-up time. A diagram may be helpful. | Methods: Study design and participants and Figure 1 |
|  | 13b | D;V | Describe the characteristics of the participants (basic demographics, clinical features, available predictors), including the number of participants with missing data for predictors and outcome. | Table 1 and sTable 1 and sTable 2 |
|  | 13c | V | For validation, show a comparison with the development data of the distribution of important variables (demographics, predictors and outcome). | sTable 2 |
| Model development | 14a | D | Specify the number of participants and outcome events in each analysis. | Table 1 |
|  | 14b | D | If done, report the unadjusted association between each candidate predictor and outcome. | N/A |
| Model specification | 15a | D | Present the full prediction model to allow predictions for individuals (i.e., all regression coefficients, and model intercept or baseline survival at a given time point). | Figure2 |
|  | 15b | D | Explain how to the use the prediction model. | Figure 2  “A ruler ranging from 0 to 100 points was scaled on top, with independent prognostic factors array on the relevant axis below. First, a subject’s age was converted to a score by drawing a straight line upward to the ruler on the top and gets the score related to age, the procedure was carried out for every covariate, and the scores obtained from every covariate were added to get the total points. |
| Model performance | 16 | D;V | Report performance measures (with CIs) for the prediction model. | AUC, calibration curve and DCA |
| Model-updating | 17 | V | If done, report the results from any modelupdating (i.e.,model specification, model performance). | N/A |
| **Discussion** | | | | |
| Limitations | 18 | D;V | Discuss any limitations of the study (such as nonrepresentative sample, few events per predictor, missing data). | Discussion: Paragraph 9  “This study also has some limitations. First, it is a retrospective study, bias is inevitable, the results should be interpreted carefully as an exploratory study. Second, since the study was carried out in a single city (Wuhan, China), the results are not fully representative. The predictive potency of the HSS score needs to be verified in other medical facilities outside of Wuhan” |
| Interpretation | 19a | V | For validation, discuss the results with reference to performance in the development data, and any other validation data. | Discussion: Paragraph 1 |
|  | 19b | D;V | Give an overall interpretation of the results, considering objectives, limitations, results from similar studies, and other relevant evidence. | Results: Paragraph 8,9 |
| Implications | 20 | D;V | Discuss the potential clinical use of the model and implications for future research. | Results: Paragraph 11 |
| **Other information** | | | | |
| Supplementary information | 21 | D;V | Provide information about the availability of supplementary resources, such as study protocol, Web calculator, and datasets. | Attached |
| Funding | 22 | D;V | Give the source of funding and the role of the funders for the present study. | N/A |

*Items relevant only to the development of a prediction modelare denoted by D, items relating solely to a validation of a prediction model are denoted by V, and items relating to both are denoted D;V. We recommend using the TRIPOD Checklist in conjunction with the TRIPOD Explanation and Elaboration document.
